# Features of the structure of psychopathological consequences in COVID-19

**DOI:** 10.1192/j.eurpsy.2022.1229

**Published:** 2022-09-01

**Authors:** N. Maruta, V. Fedchenko, I. Yavdak, O. Tkachenko, V. Zavorotnyy

**Affiliations:** 1 “Institute of Neurology, Psychiatry and Narcology of NAMS of Ukraine” SI, Borderline Pathology, Kharkiv, Ukraine; 2 Kharkiv National Medical University, Psychiatry, Narcology, Medical Psycholgy And Social Work, Kharkiv, Ukraine

## Abstract

**Introduction:**

International experience convincingly shows the prevalence of mental disorders secondary to COVID-19, the pathogenesis of which includes biological and psychosocial factors, which characterizes this area of research as relevant and timely.

**Objectives:**

Analysis of the structure of the most common forms of psychopathology within consequences in COVID-19.

**Methods:**

The study involved 45 patients with depressive episodes of varying severity (F 32.0, 32.1, 32.2) and 37 ones with anxiety disorders (F 40, 41). The average age of the examined groups was 39.42 ± 5.68 and 31.54 ± 4.36 years respectively. Clinico-psychopathological, psychodiagnostic, statistical methods were used.

**Results:**

Significantly more patients with depressive disorders before the first clinical manifestations of the disease experienced COVID-19 in mild and moderate form (31.82% and 68.18% of individuals, respectively) (p <0.05), while patients with anxiety disorders were more affected to some stressors of the SARS-COV-2 pandemic (including threatening information background (83.78% of people), quarantine measures in the form of self-isolation (75.66% of people), uncertainty of the impact of coronavirus infection on the socio-economic situation) (62.16% of people)) (p < 0.05). It was found that the examined patients with a history of coronavirus COVID-19 are more likely to have depressive and asthenic syndromes in the clinical picture (p < 0.05), while patients with psychogenic effects of the pandemic - anxiety-phobic and somato-autonomic syndromes (p < 0,05).

**Conclusions:**

Data on the influence of coronavirus disease COVID-19 and stressors of the SARS-CoV-2 pandemic on the formation of mental disorders of various genesis will allow to develop prevention algorithms and personalize therapeutic programs.

**Disclosure:**

No significant relationships.

